# Study of the Chlorine Influence on the Corrosion of Three Steels to Be Used in Water Treatment Municipal Facilities

**DOI:** 10.3390/ma16062514

**Published:** 2023-03-22

**Authors:** Rúben D. F. S. Costa, Marta L. S. Barbosa, Francisco J. G. Silva, Susana R. Sousa, Vitor F. C. Sousa, Bruno O. Ferreira

**Affiliations:** 1 ISEP, School of Engineering, Polytechnic of Porto, 4249-015 Porto, Portugal; 2 Associate Laboratory for Energy, Transports and Aerospace (LAETA-INEGI), 4200-465 Porto, Portugal; 3 i3S—Instituto de Investigação e Inovação em Saúde, Universidade do Porto, 4200-135 Porto, Portugal; 4 INEB—Instituto de Engenharia Biomédica, Universidade do Porto, 4200-135 Porto, Portugal

**Keywords:** corrosion, degradation, metals, municipal facilities, chlorine action

## Abstract

Many municipal facilities, such as pools and drinking water treatment facilities, are subject to ongoing maintenance due to the corrosion of their metallic materials caused by chlorine, leading to high costs and a possible risk to public health. A proper study of the employed product’s effect could lead to the use of better materials, which significantly increase the lifetime of metallic equipment more attacked by corrosion, through studies evaluating their cost-effectiveness. This paper was carried out with the objective of studying the degradation of some metallic materials (AISI 316L, AISI 321 and Duplex 14462) used in the referred facilities in order to select the one that possessed a better behavior. It was observed that the introduction of some more adequate materials can drastically reduce maintenance operations, with Duplex 14462 showing the best results, ideal for greater chlorine concentrations, followed by AISI 321, which may be employed for components in less contact with chlorine, since it is more easily affordable.

## 1. Introduction

Chlorine, under conditions of ambient temperature and atmospheric pressure, is an irritant gas with a greenish-yellow color and is usually sold in steel cylinders in liquid form under pressure (it becomes a liquid at 6.8 atmospheres and 20 °C) [1]. Chlorine and sodium hypochlorite (NaClO), although seemingly similar, as the two are used to disinfect water, have some differences, namely, concerning their concentrations used in the industry [2]. Chlorine is obtained by the electrolysis of a sodium chloride (NaCl) solution. The chlorine present in this solution is initially in the form of gas, turning into liquid after the temperature decreases and after compression [3]. Thus, chlorine is used as a water treatment product, either for human consumption or for swimming pools [4]. It is also employed as a raw material in the production of hydrogen chloride, hydrochloric acid, dichloroethane, and sodium hypochlorite [5,6,7,8]. On the other hand, the latter is obtained through a reaction between chlorine and a dilute caustic-soda solution. Sodium hypochlorite is mainly used in the production of disinfectants and is displayed in a liquid state, reaching a chlorine content of 10% to 15% [9].

Given the purpose meant for the water, it is given particular importance to impurities it carries, such as salts, acids, dissolved gases and suspended materials, among others, in order to prevent corrosion problems [10]. One factor to consider is the possibility of combined actions of mechanical stresses and the corrosive environment, as drive fluids exhibit accelerated corrosion by the joint action of chemical factors such as chlorine, in the case of treatment water [11]. Given that the impurities can cause the deterioration of equipment and pipes where water will circulate, this being intended for human consumption, then the factors that most often influence the corrosive action of water, such as sodium chloride, chlorine, oxygen, organic matter, suspended solids, bacteria, etc., must be considered [12]. Dissolved salts may act by accelerating or slowing down the speed of the corrosion process, with chlorine being the compound that influences this process the most [13]. The corrosion effect of sodium hypochlorite is due to the fact that this compound is a strong electrolyte and thereby causes an increase in conductivity, which, as mentioned above, is fundamental in the mechanism of electrochemical corrosion [14]. The solubility of oxygen decreases continuously with the increasing concentration of NaClO, which explains, in many cases, the reduction in the corrosion rate in high concentrations of NaClO [15].

Metallic materials are divided into ferrous and non-ferrous metals. The ferrous metals are comprised mostly of iron (Fe) and may contain small amounts of other elements such as carbon or nickel in a specific ratio, which are added to achieve desired properties. All ferrous metals are generally magnetic and have high tensile strength, with the major drawback of being susceptible to corrosive attacks with some ease [16]. Cast iron is commonly used in pipes for water supply, usually coated on the inner surface by epoxy resin [17]. The degradation of the molten irons is similar to that of normal steels; however, these have a typical form of corrosion called “selective corrosion”—more specifically, the “graphitization”, which is the disappearance of the material’s constituents, leaving only the graphite remaining [18]. In graphitic corrosion, the material remains visually unchanged while maintaining the original shape and distances, particularly by changing the mechanical properties. It is possible to observe in a cast iron and carbon steel pipe, after some time of use, the deposition with a generally brown–orange color inlay, which brings serious disadvantages in the distribution of drinking water [19].

Stainless steels are iron and chromium (Fe-Cr) alloys which have at least 10.50% Cr, allowing for the formation of an extensive set of materials through the addition of other elements. As with all steels, the stainless group also contains carbon and other elements, such as silicon (Si), manganese (Mn), phosphorus (P) and sulphur (S); however, chromium and nickel deserve to be highlighted. Chromium is an important element in corrosion resistance, whereas nickel (Ni) contributes to the improvement of the alloy’s mechanical properties. There are several classifications of stainless steels, which can be divided into two large main groups: the 400 series constitutes magnetic steels that are basically Fe-Cr (ferritic and martensitic) alloys, whereas the second one, the 300 series, is comprised of non-magnetic steel. The latter differs from the 400 series primarily by the addition of nickel, being essentially Fe-Cr-Ni, which are classified as austenitic steels. One of the problems of some stainless steels (especially the 304) is the corrosive action caused by the chloride anion (Cl^−^), depending not only on the concentration of chlorides in the middle but also on the temperature and pH. In an environment of this type, corrosion can occur in the form of pitting, cracks or stress [20]. On the other hand, ferritic steels are more prone to just pitting and cracks [21]. In general, the austenitic steels are more resistant to these two forms of corrosion because of the nickel action, which contributes to the re-formation of the passivation film material when it is broken by them [22].

The chemical stability of steels differs greatly depending on the acidic media in which they are found. The oxidizing acidic media promote the formation of the passive film, whereas the reducing acids do not allow for the formation of this film, which means that stainless steel is not recommendable in the latter case [23]. In reducing acid environments, corrosion always presents itself uniformly. In the case of the stainless steel already having a passive film, the corrosive action will cause the rupture of the film. In media containing the anion chloride, stainless steels may suffer mainly localized corrosion (for example, pitting). In this case, the corrosive attack will cause gaps in the metal passivation film [20]. The formation speed of these gaps increases with the concentration of chlorides in the medium the steel is in. The gaps are occupied by the base metal migration, thus being eliminated, and its concentration will depend on its rate of creation and elimination. In the case of the creation speed being predominant, the passive film loses cohesion and undergoes localized disruptions [24]. The addition of antioxidants to control bacteria—for example, in water treatment for human consumption—should be performed with great care.

In the case of pipes intended for water supply, the zinc galvanization involves the immersion of extruded steel tubes in molten zinc baths and has a significative presence in the facilities. Generally, this material has a roughened inner surface, resulting in an uneven deposition of zinc. Thus, some parts of the equipment are not covered, which means that the material will not be fully protected, this being one of the main sources of corrosive attacks [25]. On the other hand, if the zinc is deposited in an excessive way, adding to the surface roughness, the material can suffer from intergranular corrosion. The degradation of the galvanized steel is similar to that of the general steels; however, galvanized steel has a typical form of corrosion called “bimetallic corrosion” or “galvanic corrosion”. This type of corrosion occurs due to the high difference of potential between materials, with the zinc being a very anodic material, which may oppose to a noble material that is very cathodic. This process is aggravated when there is a high conductivity of water [26].

Non-ferrous metals are those whose constitution does not include iron (Fe). These are commonly used materials for direct contact with water due to their oxidation resistance, which is superior to that of ferrous metals. In spite of this, they are not always a good choice, because although their degradation is slower, it can be more dangerous and contaminate the water. There are several non-ferrous metals chosen in the distribution of water for human consumption, but the emphasis is on the brass, as the alloy that is most commonly used [27].

The main objective of this work is to study the corrosion problems induced by the use of chlorine in municipal facilities. Therefore, it can be considered that the objectives are to identify Chlorine-induced corrosion problems by measuring the operating time of certain components subject to degradation. This will be achieved by comparing the useful life of the equipment with the same components in other metals, as well as by carrying out adequate research and selection of materials, with a view to replace parts or the entirely previously used materials. Afterwards, some accelerated degradation tests will be performed for the materials that are intended to be used in order to decide the best alternatives.

**Figure A1:**
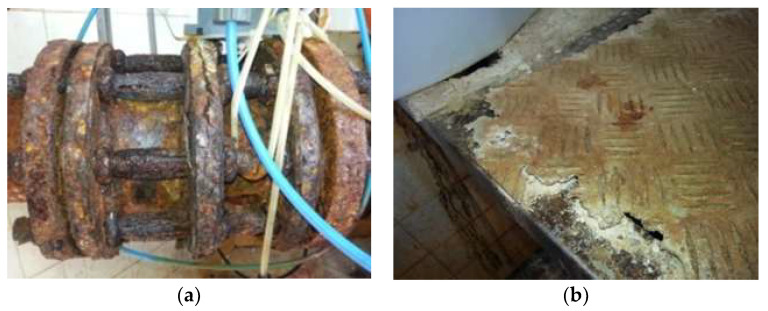
Joint screws on the reservoir water inlet pipe (**a**) and reservoir floor (**b**) uniform corrosion and in the form of cracks in the municipal facilities.

In municipal facilities, several cases of the deterioration of metallic materials due to chlorine were found, such as pipe joints screws (Figure A1a), pipes, water analyzer support panels, electrical components, valves, pipe supports, equipment and even certain reservoirs accesses (Figure A1b) involving many corrosive processes, which varied according to the NaClO concentrations used. In most cases, if this deterioration had been properly considered at the design stage, and if certain construction details had been taken into account, without necessarily implying an increase in cost, the corrosive attacks could have been avoided or at least minimized. In many cases, the studies consider the compatibility of the material and environment but often disregard possible operational or environmental changes. All these corrosive attacks checked to date show that even materials with higher costs are not immune to corrosion when they are not best suited for the purpose.

## 2. Materials and Methods

In order to achieve the objectives, it becomes necessary to:

- Conduct a survey of all components that were particularly attacked by chlorine;
- Investigate the studies conducted to date on the corrosion caused by chlorine in these materials;
- Measure the degradation time of each component and material in practice;
- Proceed with an adequate selection of materials, taking into account the requirements of each product/component;
- Analyze the comparative results of the materials’ degradation when exposed to excessive chlorine concentrations for accelerated corrosion tests;
- Study the possibility of carrying out the same functions performed by each component in alternative materials, which may offer better conditions of resistance to corrosion caused by chlorine;
- Evaluate the cost–benefit of the materials’ possible replacement.

### 2.1. Materials

The materials selection is a crucial process in the replacement of the corroded equipment. Should the choice of a material be inappropriate, it may lead to serious consequences for both the supplier and the receiving company, going from failure of the product to a huge increase in costs. The selection of materials involves a large variety of functional factors such as the project requirements, the material properties that specify these requirements, the costs and the manufacturing processes. For the study of a new process or the improvement of any existing project, there is a need to select materials in order to establish a processing relationship, a structure, properties and performance and meet the requirements of the end-consumer. To prepare this selection, the specific fields must be considered, such the chemical structure of the material, its atomic arrangement, its atomic stacking factor, its physical properties and its mechanical properties, among others, taking into account the role to be played by the final product [8]. Since this study focuses on the study of materials for contact with drinking water, a range of materials that will not harm water quality, and consequently not risk public health or that of employees due to excessive contact with the material, must be considered. Considering all these factors, the most important properties for the selection of adequate substitute metals are their corrosion resistance, the cost of the materials and manufacturing processes, the wear and impact resistance and their weight, ductility, stiffness and mechanical resistance, and the materials must be inert to avoid any possible reaction with the chloride.

Within a huge range of metallic materials, from the performed selection of materials, the chosen ones were the stainless steels AISI 316L and AISI 321, as well as the Duplex 14462. The reasons behind this selection are that, due to the added alloying elements in each of them, a high corrosion resistance is achieved, especially a great resistance to pitting corrosion, one of the main causes of degradation in stainless steels. They also possess good mechanical resistance.

### 2.2. Methods

This experimental study was performed by a stagnant immersion corrosion test, as it is a simple and efficient method of corrosion testing. Generally, it is in this type of test that a higher degree of corrosion can be achieved in a short period of time, this being variable with the type of material and solution used. This test is widely used when rapid responses are needed, and, in a similar way, as for any other type of accelerated corrosion test, the intention is always to evaluate the samples by comparison, with the finality of obtaining better interpretations of the results.

Initially, the chemical composition of the metallic materials used in this study was analyzed by the spectrometry method. The objective of this analysis was to observe the possible chemical changes suffered by the metallic materials after their immersion during the stipulated periods of time (three weeks and three months). Afterwards, all samples were weighed in order to evaluate the future weight gain or loss, according to each case.

For each metallic material, five vessels were used, containing dilutions of 2%, 5%, 25%, 50% and 100% of NaClO, concentrations in % (*v*/*v*). Additionally, 10 samples with small dimensions (30 mm × 20 mm) and 4 samples of larger dimensions (140 mm × 20 mm) were prepared. Two small samples were placed in each vessel, and two larger samples were added in both vessels with dilutions of 5% and 100% sodium hypochlorite. Half of the samples had a test time of three weeks, and the other half was only withdrawn three months after the solution immersion. Each type of metal had the following variables: the immersion time, the chlorine concentration, and the samples’ dimensions (small or large).

The sodium hypochlorite solutions were prepared, with the distribution of the samples being that referred to before. The tests were carried out at 21 °C, and the temperature was kept constant throughout their entire duration. Then, the containers were closed with a transparent film with small holes so that the possible gases produced could be released.

Several methodologies were chosen for analyzing the results of the tests, and the following methods were adopted:

- Visual inspection: Through the naked eye, this method allowed for the observation of the samples before their withdrawal from the respective vessels, while they were being removed, as well as after drying and cleaning. After a first evaluation, moving distilled water was used to wash the samples so that possible mixtures of corrosion products between the different materials could be avoided. This procedure had the purpose of removing impurities. Subsequently, the samples were subjected to room temperature (about 25 °C) for two days to dry, without being in contact with other materials, with the intention of preventing any removal of the base material;
- Mass variation: The mass variation of the samples was calculated through a Denver Instruments APX-200 Precision Analytical Balance, with the initial mass of each dry sample used as a reference for the mass after the accelerated corrosion tests;
- Optical microscopy: The method was carried out with an OLYMPUS model BX51M microscope. For the optical microscopy analysis of the metallic materials under study, fifty times (50×) and one hundred times (100×) magnifications were used. In order to allow for the observation of the degradation’s variation, two samples of each type of material, immersed in solutions containing 2% and 100% of sodium hypochlorite, were analyzed to evaluate the difference between a low and a high corrosion influence;
- Scanning electron microscopy: For this evaluation, an FEI microscope, model Quantum 400, equipped with an EDAX system was chosen for it to be able to perform micro-analyses by energy dispersion. For the analysis of the morphology of the materials by scanning electron microscopy, only the immersed samples in a 100% concentration of sodium hypochlorite were used, because these were the ones that suffered the greatest degradation;
- Characterization of chemical composition through spectrometry: For this study, the chemical composition tests were performed with the aid of an optical emission spectrophotometer of the SPECTRO brand, model SPECTROLAB M8. After an analysis carried out on samples of 316 L stainless steel, stainless steel 321 and Duplex 14462, without being dipped in NaClO solution, a Mass Spectroscopy analysis was performed on the samples subjected to 100% of NaClO immersion for three weeks and three months. The objective of this procedure was to compare the differences between both groups under a great chlorine concentration in terms of chemical composition, that is, how elements were brought to the sample and how others were reduced or eliminated from it;
- Mechanical test (Tensile test): The tensile tests were performed by a universal tensile testing machine of the brand SHIMADZU, model Autograph AG-X 100 kN “. In this work, uniaxial tensile tests were carried out in four samples of each metallic material, immersed in different solutions (5% and 100% of NaClO, both extremities, so that the influence of the chlorine concentration was assessed) and for different times (three weeks and three months), as well as in a standard sample for comparison. In a first step, the samples were identified and marked, with their useful length limits traced (in this case, two points were marked between claws at 90 mm), with the objective of the material’s elongation being later evaluated. Afterwards, the specimen was tightened in the machine’s gripper, this being the program set up for the tensile test. The tests were performed only with the large samples (140 mm × 20 mm) and at room temperature. The displacement velocities were kept constant: 4 mm/min, according to the ISO 6892-1 2009 standard. Finally, the evaluated samples were compared to a standard sample of each material, and an analysis of each sample’s breaking zone was conducted. The main purpose of this study was the observation of the immersed materials’ mechanical behavior in two different solutions of sodium hypochlorite without any preparation of the sample and using significantly higher parameters when compared to the real situations used in drinking water treatment facilities and swimming pools.

The results in terms of mass and mechanical strength are the average values obtained regarding the number of samples tested, as previously mentioned. The standard deviation values reported were always below 5%, showing a good repeatability in all tests (mass and tensile tests).

## 3. Results and Discussion

### 3.1. Mass Spectrometry

The analysis of the samples of AISI 316L, AISI 321 and Duplex 14462 showed that they possess a high percentage of iron, chromium and nickel, but also some molybdenum and magnesium in the case of the former and the latter. The presence of chemical elements such as phosphorus, sulphur and tin was also observed in the analyses carried out on the three metallic samples, which can be harmful to them. Some of these elements, mainly tin, are present possibly due to contaminations that occurred during the material manufacturing process.

An element also observed in the three metallic materials under study was nitrogen. This element is added to steels with a high chromium content, characterized as high chromium-manganese stainless steels, which also have molybdenum, silicon, niobium and vanadium. These have high resistance to high temperatures, as well as low carbon contents, which means that no transformation occurs at these temperatures.

The mass spectrometry analysis of the samples is crucial as a comparison method after the accelerated corrosion tests are made for the understanding of the variation in their chemical composition due to the corrosion caused by the chlorine. Only with this information is it possible to know the agents behind each one of the different types of corrosion that occurred in each sample.

### 3.2. Phenomena Observed after the Immersion of the Samples

Two days after being immersed in the solutions, all materials showed some gas liberation, which was higher with the increase in the NaClO concentrations and was caused by the reaction between the materials and the solution; however, no change in the shape or coloration in the solutions was registered.

**Figure 1:**
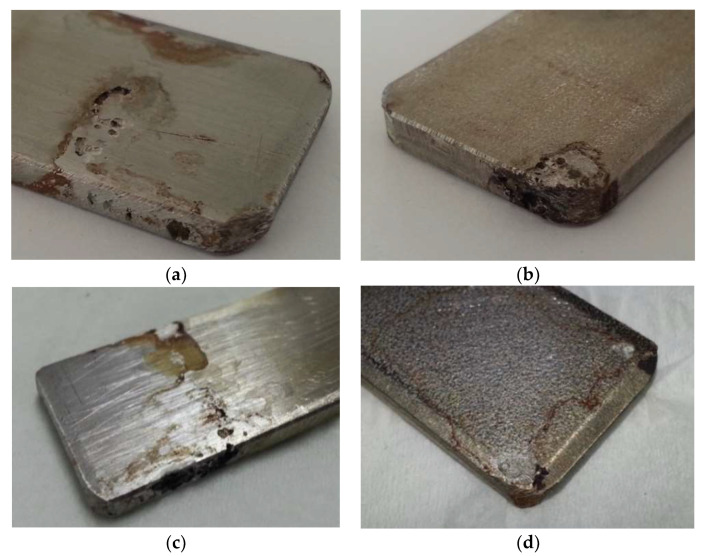
Dry samples of AISI 316L and AISI 321 after three weeks (**a**,**b**) and three months (**c**,**d**).

After three weeks of being immersed, the solutions changed in coloration to a pale yellow, a phenomenon that is explained by the beginning of the corrosion in the materials. Although the Duplex 14462 did not show any signs of being affected by the NaClO, the AISI 316L and AISI 321 have shown visible signs of accentuated corrosion, as well as a significant amount of corrosion product in the bottom of the containers. The samples were then removed from the vessels and washed. Afterwards, a visual evaluation was carried out, revealing localized pitting, as seen in Figure 1a,b.

After three months, the Duplex 14462 remained unaltered, presenting a great resistance to corrosion. On the other hand, the two stainless steels presented a greater advance in the degradation in comparison with the three weeks of immersion, as observable in Figure 1c,d, showing the other side of the samples, which was perfectly clean before.

### 3.3. Mass Variation Analysis after Three Weeks and Three Months

**Figure 2:**
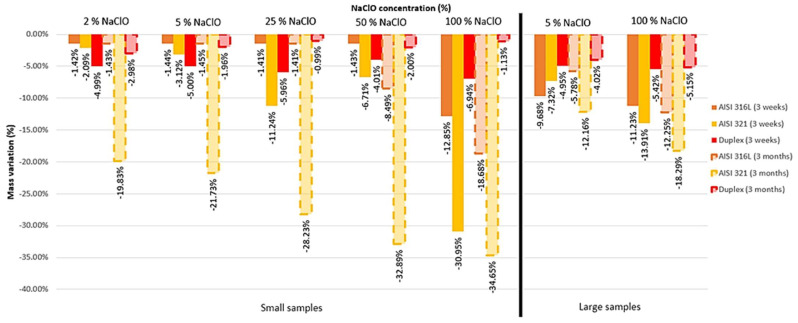
Mass variation of all the samples after three weeks and three months of immersion.

In Figure 2, the mass change suffered by the small and large samples after three weeks and three months is presented.

After three weeks of immersion, by analyzing the small samples, it can be concluded that there was a greater variation in mass for 25% and 100% NaClO, the latter being more accentuate, which proves that a high concentration of the disinfection solution causes greater corrosion. The AISI 321 stainless steel was the one that lost more mass, and the Duplex 14462 was the least affected by the degradation and, therefore, the one that reacts better to the attack. On the other hand, after three months, as the NaClO concentration increased, the mass loss of the AISI 321 became greater, which was strongly affected by the corrosion. The AISI 316, despite having higher strength, also showed significant mass loss. Only the Duplex 14462 showed good resistance to degradation, having registered a reduced mass variation better than that after three weeks of immersion.

As for the large samples, after three weeks, the Duplex 14462 suffered a reduced mass variation, while the AISI 321 was greatly affected by the solution, leading to a greater loss of material. Although in low concentrations the AISI 321 steel suffers less mass variation than the AISI 316L, when the concentration increases, it is the former that suffers more mass loss. Three months following the immersion, the AISI 316L, unlike that at three weeks, suffered a low mass variation for 5% NaClO but was more affected for 100% NaClO. The AISI 321 continues to be the most affected and the Duplex 14462 continues to be the most resistant to the corrosive action of the solution, although it did not have as good results as in the previous case, meaning that it resists corrosion better in the long term with small samples.

### 3.4. Optical Microscopy Analysis of Surfaces

In order to allow for the observation of the degradation variation, two samples of each type of metallic material, immersed in solutions containing 2% and 100% of sodium hypochlorite, were analyzed by optical microscopy (OM), with magnifications of 50× for the AISI 316L and 100× for the AISI 321 and Duplex 14462. These magnifications were selected because they are the most perceptible ones in observing the corrosion evolution in each sample. In spite of two different concentrations having been observed, only the 100% NaClO will be analyzed in order for it to be possible to evaluate the most extreme corrosion effects of the solution in the metal samples.

#### 3.4.1. AISI 316L

**Figure 3:**
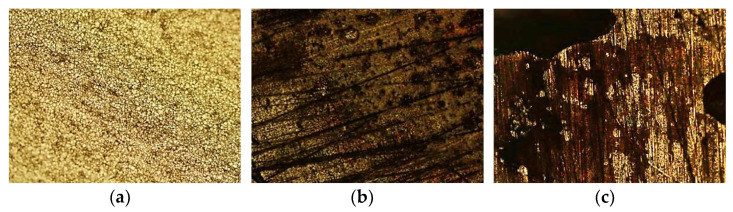
Optical microscope images of the AISI 316L samples at a magnification of 50×: (**a**) before immersion, (**b**) after three weeks, (**c**) after three months.

Figure 3 shows the AISI 316L sample, with a 50× magnification, without having been in contact with the solution (a), after three weeks (b) and after three months (c).

In Figure 3a, it is possible to observe the grains of the sample, as well as the absence of degradation. In (b), the appearance of dark areas is evident, resulting from the beginning of the corrosion process, which increases drastically in (c), after three months, with a corroded area in the upper-left corner of this image that started as small cracks and evolved until the visible size.

#### 3.4.2. AISI 321

**Figure 4:**
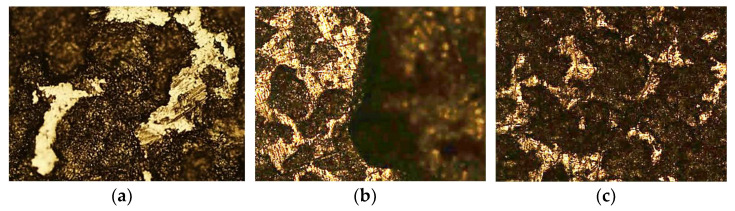
Optical microscope image of the AISI 321 sample at a magnification of 50×: (**a**) before immersion, (**b**) after three weeks, (**c**) after three months.

In Figure 4, it is possible to observe the AISI 321 sample, with a 100× magnification, before immersion (a), after three weeks (b) and after three months (c).

Contrary to the previous stainless steel, the AISI 321 before immersion (a) presents more areas of dark coloration than the white ones, indicating the presence of some oxides in the sample. In image (b), after three weeks, a high corrosion level is visible, with its right side completely degraded. After three months (c), the dark areas increased in size and became more blurred, a distinct sign of the greater level of corrosion, started by pitting.

#### 3.4.3. Duplex 14462

**Figure 5:**
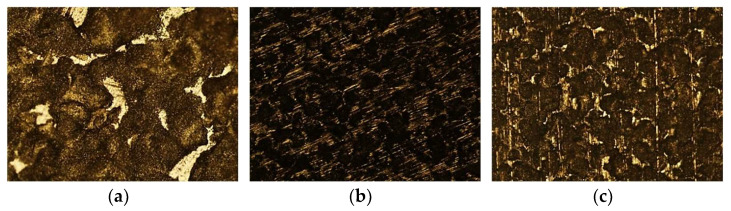
Optical microscope image of the Duplex 14452 sample at a magnification of 100×: (**a**) before immersion, (**b**) after three weeks, (**c**) after three months.

The Duplex 14462, also with a 100× magnification, had a better behavior than the first two alloys; however, the chlorine’s effect can be observed. Figure 5 shows the sample before contact with the solution (a), after three weeks (b) and after three months (c).

With a microstructure comparable only to the AISI 321 before the immersion, as seen in Figure 5a, after three weeks, the Duplex 14462 shows darker areas, whereas previously, there were clear interstices, which constitute evidence of the beginning of the degradation process; however, these are not intense. Even after three months, the Duplex 14462 presents a much better result than the previous steels, indicating that, among the three, it has the best resistance to the corrosion effect of the NaClO solution.

### 3.5. Electron Microscopy Analysis of Surfaces

For the analysis of the materials’ morphology by scanning electron microscopy (SEM), only the samples immersed in 100% concentration of sodium hypochlorite were used, considering that these were the ones that suffered the greatest degradation. In this analysis, only the samples were evaluated, and not the degradation products after immersion tests, due to the fact that the products formed were solubilized.

#### 3.5.1. AISI 316L

**Figure 6:**
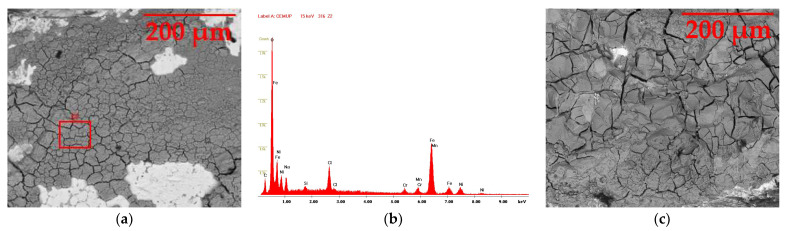
SEM image of the AISI 316L sample at a magnification of 500×: (**a**) after three weeks, (**b**) EDS of the red zone in the first image and (**c**) after three months.

**Figure A2:**
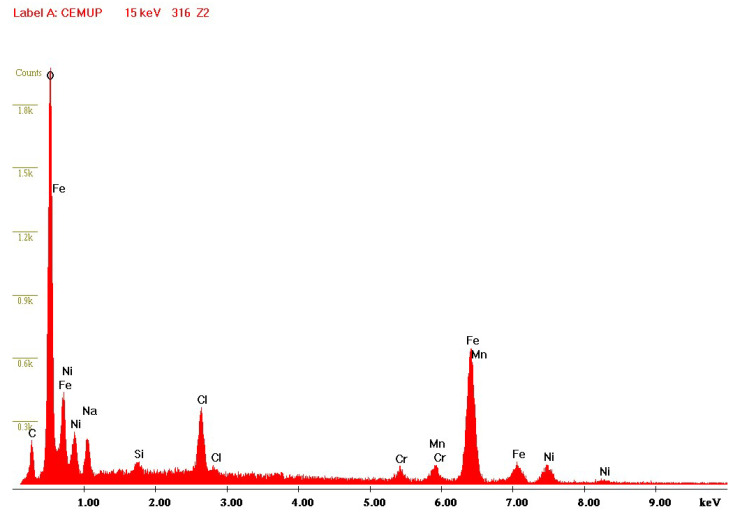
EDS of the red zone in the first AISI 316L SEM image.

The AISI 316L stainless steel sample in the SEM is presented in Figure 6 at a 500× magnification in two situations: after three weeks of immersion (a) and after three months of immersion (c). Image (b) shows the EDS graphic of the zone in the red square in (a) and can be consulted in Figure A2.

In image (a), the majority of the sample’s surface is covered in cracks, which is representative of the start of its degradation. In spite of this, there are also some white-colored areas, which means that not all of the metal was affected by the solution. As for the chemical composition, the white areas, which were less affected, kept small amounts of iron, chromium and nickel, as they reacted with the oxygen and formed a protective layer of chromium oxide, as visible in image (b). Nevertheless, no traces of molybdenum nor magnesium were found, since they were destroyed with the NaClO. In addition, the grey area shows traces of oxygen, due to the corrosion, as well as sodium and chlorine from the solution. On the other hand, in image (c), there are almost no white areas, as the corrosion was more advanced, which is also visible through the increase in the cracks’ size, indicative of a higher state of corrosion after three months.

#### 3.5.2. AISI 321

**Figure 7:**
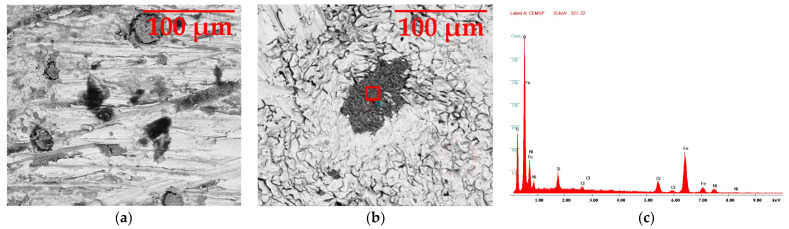
SEM image of the AISI 321 sample with 1000× magnification: (**a**) after three weeks and (**b**) after three months. (**c**) EDS image of the red zone in the second image.

**Figure A3:**
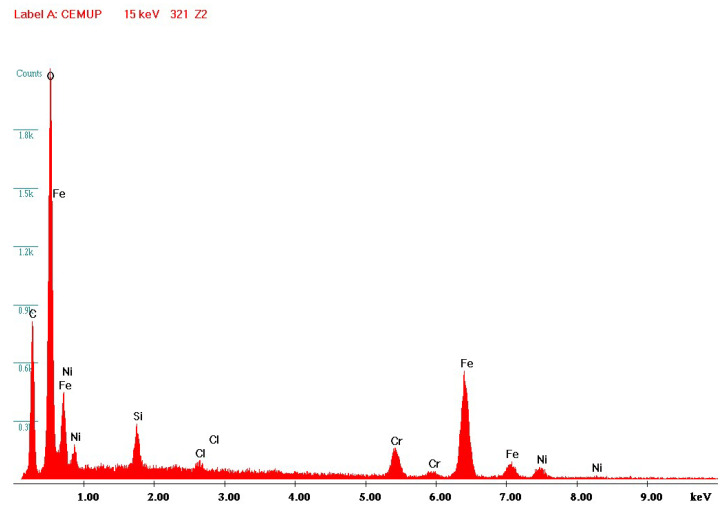
EDS of the red zone in the second AISI 321 SEM image.

Figure 7 shows the AISI 321 stainless steel sample in the SEM with 1000× magnification in two situations: after three weeks (a) and after three months of immersion (b). In the second image, the corrosion effect was greater, as visible through the increase in the circular pitting size and the darker color in its center. Image (c) shows the EDS graph of the darker area of image (b), highlighted through a red square and present in Figure A3.

In contrast to the AISI 316L, the AISI 321 stainless steel presented more distinct signs of corrosion already after three weeks (image (a)), proving its lower resistance to degradation. The irregular darker circles mean that, in this steel, the corrosion has propagated in the form of pitting, unlike the cracks in the previous one. In image (b), the evolution of the corrosion is highlighted, with the circular zones which started as pitting having increased in their dimensions, visible through the dark area in the center, as well as developed fissures in the surrounding zones, an additional defect. The dark area, as seen in image (c), possesses constituents such as oxygen and chlorine, representative of the corrosion, and the white area, although having been less affected, also has some of these chemical elements but also maintains several of the initial constituents: iron, chromium and nickel.

#### 3.5.3. Duplex 14462

**Figure 8:**
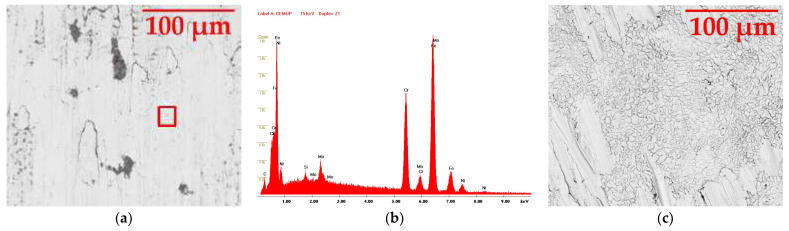
SEM image of the Duplex 14462 sample: (**a**) after three weeks with 500× magnification, (**b**) EDS of the red zone in the first image and (**c**) after three months with 1000× magnification.

**Figure A4:**
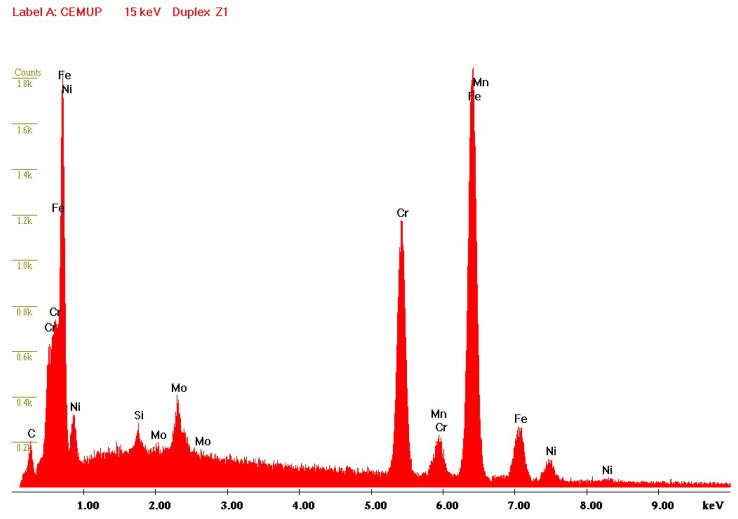
EDS of the red zone in the first Duplex 14462 SEM image.

The Duplex 14462, on the other hand, presents a completely different result from the previous two samples; it is much more resistant to corrosion caused by the chlorine. Figure 8 represents the sample with a 500× magnification after three weeks of immersion (a) and with a 1000× magnification after three months of immersion (c). Different magnifications were used to observe the influence of the chlorine in more detail. Image (b) shows the EDS graph of the red-highlighted zone in image (a), which can be consulted in more detail in Figure A4.

The Duplex 14462 samples confirm what had already been demonstrated by optical microscopy: this material had a superior corrosion resistance compared to that of the other two; however, it still had some small grey zones affected by the solution, as seen in image (a), with the predominance of oxygen and chlorine, the origin of the corrosive attacks. Nevertheless, the white areas, making up the majority of the image, kept the concentrations of chromium, nickel and molybdenum present in image (b), showing that there was no deterioration. Image (c) corroborates the practical immunity of the Duplex 14462, with only some scratches on its surface, caused during the cleaning stage.

### 3.6. Tensile Tests

After the microscopic analyses, experimental tests of uniaxial traction were performed. The specimens used in the tests did not suffer any pre-treatment, so it was possible to recreate real service conditions in contact with chlorine.

#### 3.6.1. AISI 316L

**Figure 9:**
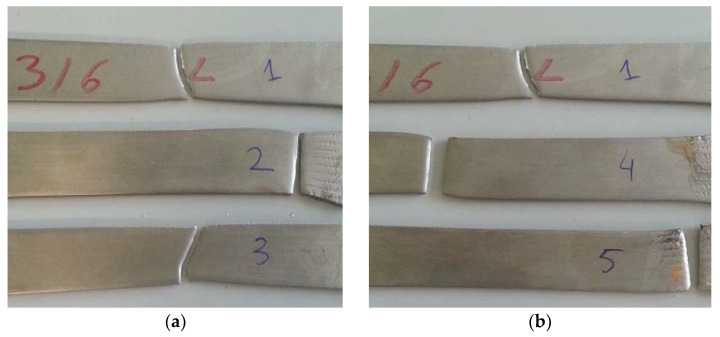
AISI 316L samples at three weeks (**a**) and three months (**b**) following the tensile test.

In Figure 9, the AISI 316L samples are shown after the tensile test, with the standard at the top, that immersed in 5% NaClO at the center and that immersed in 100% NaClO at the bottom, at three weeks (a) and three months (b) of immersion.

After three weeks (a), the standard specimen and the one immersed in 100% NaClO ruptured approximately in the same zone, in the center, while the specimen immersed in 5% NaClO ruptured near the clamping area of the clamps of the testing machine. On the other hand, after three months, both samples in contact with NaClO ruptured near the clamping of the test machine claws, a fact explained by a significant difference in the cross-sectional area near the rupture zone, due to the corrosion suffered. The standard did not show this behavior, presenting a uniform cross-sectional area throughout its length. In the first case, the third sample presented a rupture similar to the standard one.

**Figure 10:**
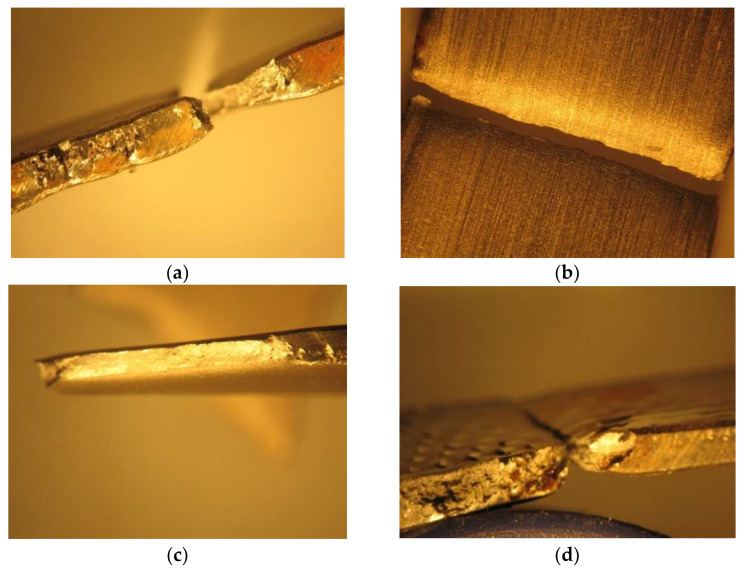
Fracture cross-section of the AISI 316L samples immersed in 5% NaClO and 100% NaClO for three weeks (**a**,**c**) and three months (**b**,**d**).

Figure 10 highlights the fracture zone of the samples immersed in 5% NaClO and 100% NaClO after three weeks ((a) and (c)) and three months ((b) and (d)).

The sample immersed in 5% NaClO for three weeks (a) presents corrosion in its lateral area, a factor that reduced the strength of the material in this region, leading to its failure closer to the clamping zone. In spite of this, after three months (c), the same sample shows no corrosion in the useful area subjected to traction, meaning that this only occurred in its exterior. As for the sample in 100% NaClO, in both cases, the rupture occurred near their edge, since these were more affected by the chlorine. After three weeks (b), no corrosion is found in the useful area subjected to traction; however, after three months (d), a pitting corrosion is clearly observable.

**Figure 11:**
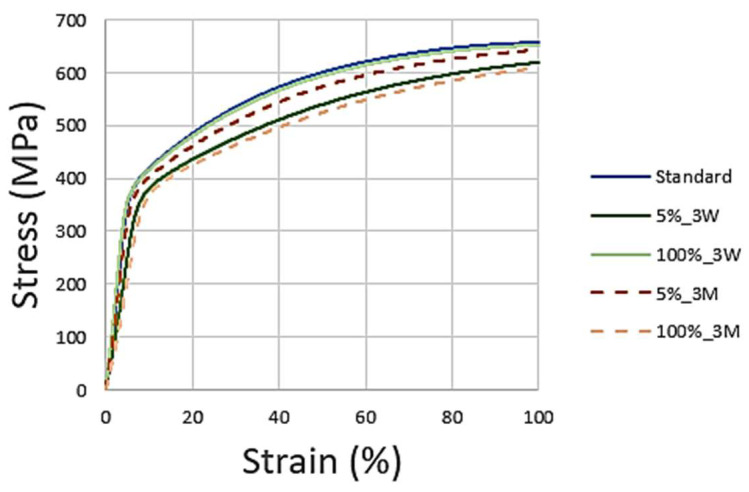
AISI 316L stress–strain curves after three weeks and three months.

Figure 11 presents the stress–strain curves for all the AISI 316L samples.

The AISI 316L presented signals of a decrease in its mechanical resistance to traction due to the NaClO effect. As expected, the standard specimen was the one with the best mechanical resistance, and the one immersed in 100% NaClO for 3 months had the worst result. The specimen at a 100% concentration for 3 weeks showed better mechanical resistance than the one at 5% for 3 months, suggesting that, in this material, the exposure time has more influence than the concentration of the reagent.

Table 1 presents the values reached by the samples after three weeks and three months at the point of maximum force as a function of the cross-sectional area. The obtained results are in line with what was previously concluded by the graph, i.e., the samples affected by NaClO corrosion present maximum strength and displacement values lower than those of the standard sample, since their properties were negatively conditioned by the solution. After three weeks, among the two concentrations, the 100% NaClO solution presented the worst results, as expected, given the greater contact with the corrosive agent. As for the three months, the 100% NaClO sample, having suffered greater corrosion, presents the lowest displacement and maximum force reached during the test. The 5% NaClO sample, although better, also presented a lower displacement and maximum force than the standard sample, demonstrating that it was not immune to the corrosive attack.

#### 3.6.2. AISI 321

**Figure 12:**
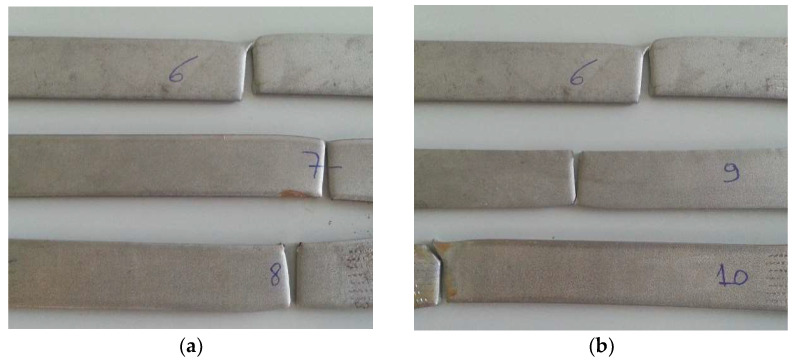
AISI 321 samples at three weeks (**a**) and three months (**b**) after the tensile test.

As for the AISI 321, Figure 12 shows the three specimens at three weeks (a) and three months of immersion (b) after the tensile test: standard sample at the top, 5% NaClO in the middle and 100% NaClO at the bottom.

After three weeks (a), although the failure suffered by the two samples immersed in sodium hypochlorite was not exactly in the same place, it was in the same region, i.e., near the clamping area of the clamps of the testing machine. As for the standard sample, it broke closer to the center, but there is not much difference between the three. In the samples after three months (b), while the standard broke in the central area, the 100% NaClO one broke near the edge, showing signs of corrosion in that region, which reduced its mechanical strength. The 5% NaClO sample fractured in an intermediate zone between the other two, but closer to the center, indicating that it was not so affected by corrosion.

**Figure 13:**
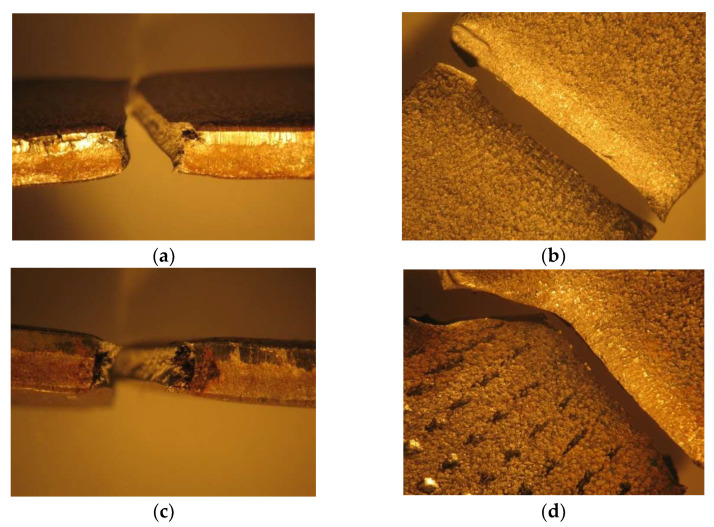
Fracture cross-section of the AISI 321 samples immersed in 5% NaClO and 100% NaClO for three weeks (**a**,**c**) and three months (**b**,**d**).

In Figure 13, the detail of the rupture zone in the two immersed samples is shown: 5% NaClO and 100% NaClO after three weeks ((a) and (c)) and three months ((b) and (d)).

The results for the three-weeks sample are similar to those of the AISI 316L, since the 100% NaClO sample (c) suffered more corrosion due to the higher concentration, but the 5% NaClO sample (a) was not immune either, because the rupture occurred near the end of the specimen. For the three months, the 5% NaClO (b) did not present visible signs of corrosion in the fracture zone, which was linear, but as this occurred somewhat away from the center, there was some degradation, although not significant. The 100% sample (d), on the other hand, presents a non-linear rupture, with visible signs of degradation.

**Figure 14:**
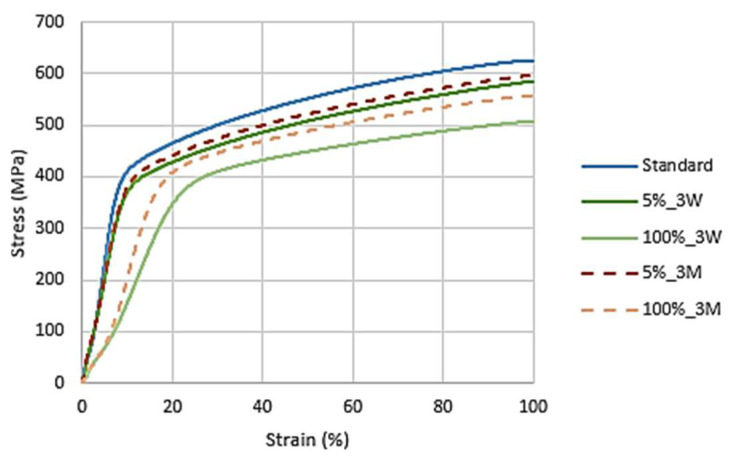
AISI 321 stress–strain curves after three weeks and three months of immersion.

Figure 14’s graph represents the stress–strain curves for all the AISI 321 samples.

Similar to what happened in the previous material, the AISI 321 also showed signs of a mechanical traction resistance decrease due to the effect of NaClO. As expected, the standard was the specimen that presented the best mechanical resistance, and the ones immersed in 100% NaClO had the least favorable results.

Table 2 shows the values reached in the maximum force by this material after three weeks and three months. Both situations confirm the observations made by the respective graphs. Compared to the AISI 316L results, the AISI 321 had a higher displacement difference between the standard and the samples immersed in the solution, which means that it was more altered by the corrosion, with worse results, as its properties were negatively affected, especially when in contact with the 100% NaClO solution.

#### 3.6.3. Duplex 14462

**Figure 15:**
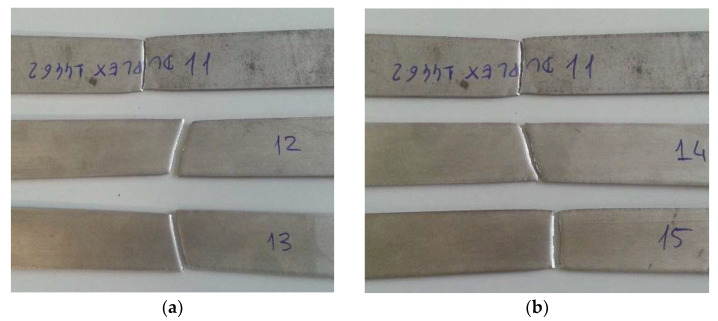
Duplex 14462 samples after three weeks (**a**) and three months (**b**) of being subjected to the tensile test.

The Duplex 14462 samples after the tensile test are presented in Figure 15. The standard sample is at the top, that immersed in 5% NaClO is at the center and that immersed in 100% NaClO is at the bottom, at three weeks (a) and three months (b) of immersion.

In contrast to the first two alloys, in this case, the samples broke in the same zone, their center, confirming that the solution almost did not influence the Duplex 14462’s properties, disregarding the concentrations used or the immersion times.

**Figure 16:**
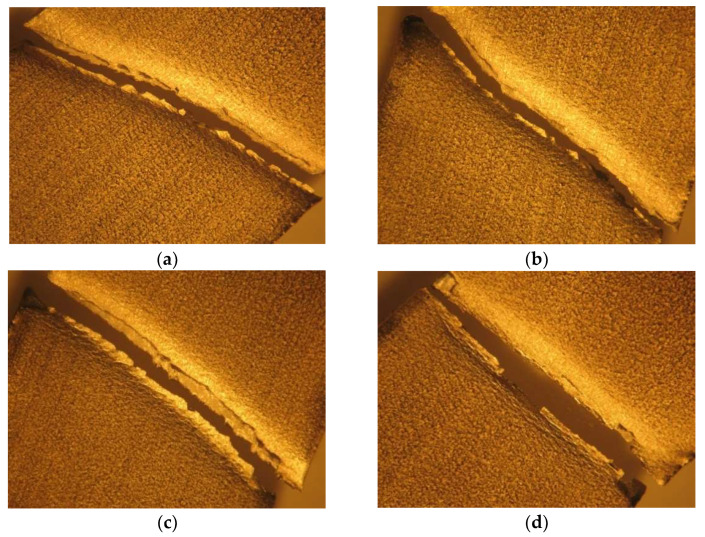
Detail of the fracture zone of the Duplex 14462 samples immersed in 5% NaClO and 100% NaClO for three weeks (**a**,**c**) and three months (**b**,**d**).

In Figure 16, the fracture zone of the samples immersed in 5% NaClO and 100% NaClO after three weeks ((a) and (c)) and three months ((b) and (d)) are presented.

**Figure 17:**
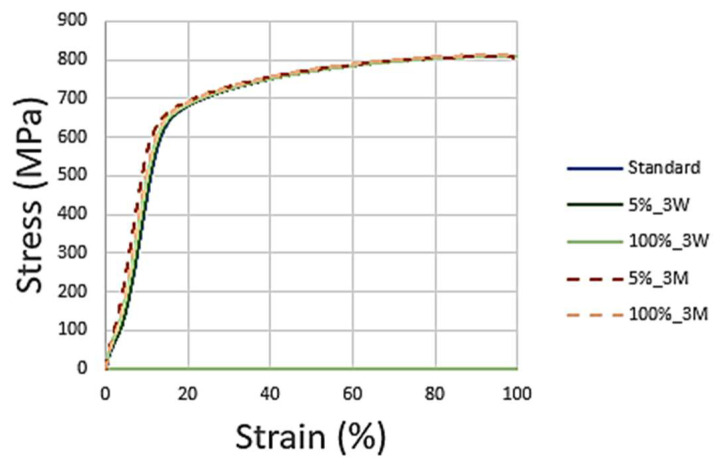
Duplex 14462 stress–strain curves after three weeks and three months.

As can be seen, the two samples in both cases show a similar fracture between each other and compared to the standard one, showing no evidence of corrosion in the useful areas of rupture and, therefore, maintaining the strength of the material. This is also observed in Figure 17, showing the stress–strain curves of the tensile tests. Only the sample immersed in 100% NaClO for three months (d), which had the most extreme conditions of the four shown, has a slight wear of the material in the fracture zone.

The mechanical resistance to the traction of Duplex 14462 showed no signs of decrease, with the curves of the tests overlapped with the standard, corroborating the previous results obtained, such as the reduced mass variation of this material. This indicates that the referred material showed negligible corrosion degradation in the most aggressive conditions tested: 100% NaClO after three months of immersion.

Table 3 represents the values reached by the samples of this material in the tensile test, proving the analysis made through the respective graphs. The samples immersed in NaClO showed maximum force and elongation values similar to those of the standard, meaning that the material was immune to corrosive attacks by the NaClO solution, both after three weeks and three months of immersion.

## 4. Conclusions

The study of alternative materials to those affected by corrosion caused by chlorine was carried out with affordable materials, so it is possible for any entity to make the replacement of their equipment without difficulty, taking into account the type of accessories or components required. There was no preparation before the evaluations in order to simulate real operating conditions, where the materials are in direct contact with several solutions of NaClO. Within the metals, three different materials were studied: the AISI 316L and AISI 321 stainless steels and the Duplex 14462.

The AISI 316L stainless steel, when immersed, gave rise to corrosion products, although in negligible quantity, and maintained strength over time, but it lost ductility. The stainless steels containing molybdenum showed a greater resistance to NaClO than those containing titanium.

As for the AISI 321 stainless steel, it has a higher price and presented the worst behavior when immersed in the solution, having suffered corrosion, which caused the loss of mechanical resistance and ductility. Hence, it is not a good solution for the intended purpose.

Finally, the Duplex 14462 stainless steel, as opposed to the others, practically did not present corrosion, having maintained the mechanical resistance and elongation capacity over time when immersed in the NaClO solution. This metal is the one with the best global result and also has a greater resistance to impact, but it is not suitable for certain applications, since it is restricted to manufacturing processes that allow for its molding or the use of standard formats, such as plates, tubes and rods.

In summary, for low concentrations of NaClO, due to the high cost of Duplex 14462, and considering the good results also obtained by the other steels, the most suitable for the applications in question is AISI 316L. Nevertheless, for high concentrations of sodium hypochlorite, Duplex 14462 is the material of choice, presenting clear advantages over the others, namely, its high long-term corrosion resistance. Therefore, this paper contributed new findings to determine what the best solution is for the material selection in metallic equipment in a chlorine-filled environment, considering the situations.

In spite of the good results, this study had limitations, such as the lack of advanced equipment for performing more complex corrosive rate measurements, this being one of the pathways to future works.

## Figures and Tables

**Table 1:** Values reached by the AISI 316L after three weeks and three months at maximum force.

Values Achieved by the Material at Maximum Force				
Immersion Time	Sample	F_max_ (N)	σ_max_ (MPa)	Displacement (mm)
	Standard	39,382.7	656.38	54.49
3 weeks	5% NaClO	39,052.5	650.88	53.48
3 months		38,558.8	642.65	45.39
3 weeks	100% NaClO	37,106.9	618.45	36.24
3 months		36,480.2	608.00	31.72

**Table 2:** Values reached by the AISI 321 after three weeks and three months at maximum force.

Values Achieved by the Material at Maximum Force				
Immersion Time	Sample	F_max_ (N)	σ_max_ (MPa)	Displacement (mm)
	Standard	50,058.7	625.73	46.71
3 weeks	5% NaClO	46,723.5	584.04	34.87
3 months		47,667.9	595.85	36.23
3 weeks	100% NaClO	40,613.3	507.67	16.98
3 months		44,632.3	557.90	25.21

**Table 3:** Values reached by Duplex 14462 after three weeks and three months at maximum force.

Values Achieved by the Material at Maximum Force				
Immersion Time	Sample	F_max_ (N)	σ_max_ (MPa)	Displacement (mm)
	Standard	32,414.1	540.24	29.97
3 weeks	5% NaClO	32,403.1	540.05	29.98
3 months		32,299.3	538.32	29.58
3 weeks	100% NaClO	32,383.0	539.72	30.08
3 months		32,546.4	542.44	29.56

## Data Availability

Not applicable.
